# Metastases from renal cell carcinoma presenting as gastrointestinal bleeding: two case reports and a review of the literature

**DOI:** 10.1186/1471-230X-7-4

**Published:** 2007-01-31

**Authors:** Gareth J Sadler, Mark R Anderson, Mark S Moss, Paul G Wilson

**Affiliations:** 1 Department of Gastroenterology, City Hospital, Dudley Road, Birmingham B18 7QH, UK; 2 Department of Radiology, City Hospital, Dudley Road, Birmingham B18 7QH, UK

## Abstract

**Background:**

Bleeding from small bowel neoplasms account for 1–4% of cases of upper gastrointestinal haemorrhage. Renal cell carcinoma constitutes 3% of all adult malignancies and often presents insidiously. Consequently 25–30% of patients have metastases at the time of diagnosis. Gastrointestinal bleeding from renal cell carcinoma metastases is an uncommon and under-recognised manifestation of this disease.

**Case Report:**

In this report we describe two cases of gastrointestinal bleeding from renal cell carcinoma metastases – in one patient bleeding heralded the primary manifestation of disease and in the other signified recurrence of disease following nephrectomy.

**Conclusion:**

These cases highlight the importance endoscopic vigilance in cases of undiagnosed upper gastrointestinal haemorrhage, especially in patients with a past history of renal cell carcinoma.

## Background

It is estimated that in 7–25% of patients who undergo endoscopy for suspected upper gastrointestinal haemorrhage, no source of bleeding is identified [1]. Malignant causes of upper gastrointestinal bleeding account for 1–4% of cases. Tumours of the small intestine are uncommon, comprising 0.35% of all malignancies [1], and symptoms are frequently non-specific. Consequently diagnosis is often difficult and may be delayed. Indeed, less than 50% of small bowel lesions are considered surgically resectable at diagnosis [2]. Thorough endoscopic evaluation of the upper small intestine and a high index of suspicion are vital for correct diagnosis and appropriate management of these lesions.

Renal cell carcinoma (RCC) behaves unpredictably and has a diverse range of clinical manifestations. Patients often present insidiously with vague abdominal symptoms – the classic triad of haematuria, loin pain and abdominal mass is found in only 4–17% [3,4]. Accordingly, 25–30% of patients are found to have metastases at diagnosis. A further 30–50% of patients with local disease will develop metastases during the course of their illness [5].

Metastases to the pancreas and small intestine in RCC are rare, but can present as gastrointestinal bleeding [6,7]. We describe two cases of pancreatico-duodenal metastasis in RCC presenting with upper gastrointestinal haemorrhage – in one case bleeding was the first manifestation of disease, and in the other bleeding heralded disease recurrence.

## Case presentation

### Case 1

A 67 year-old male with a past history of angina and arthritis presented with a one week history of melaena and lethargy. He was found to be anaemic (Hb 6.7 g/dl, MCV 92.2 fl). Endoscopy revealed moderate haemorrhagic gastritis and a Campylobacter-like organism (CLO) -positive duodenal ulcer, but no signs of recent bleeding. His non-steroidal anti-inflammatory medication was stopped and eradication therapy prescribed. He remained an in-patient for one week, during which time seven units of blood were transfused to correct his Hb to 11.6 g/dl.

Six weeks later he presented with abdominal pain and further melaena requiring a 2-unit blood transfusion. Endoscopy revealed mild antral gastritis and a normal duodenum but no source of bleeding. Colonoscopy was normal. Two weeks later endoscopy was repeated which showed a small amount of fresh blood in the duodenal bulb, but no obvious lesion was seen to account for the bleeding.

**Figure 1:**
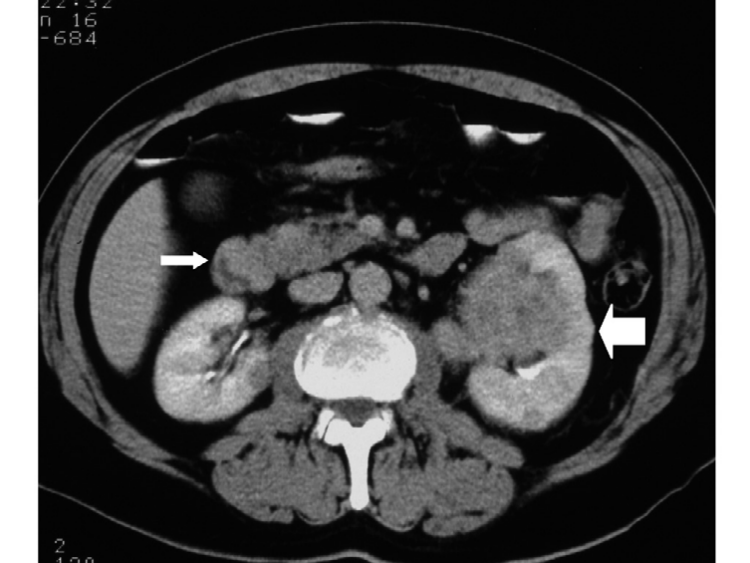
**Abdominal CT scan**. Abdominal CT scan demonstrating a left-sided renal tumour (thick arrow) and a polypoidal mass in the wall of the second part of the duodenum (thin arrow) arising from the pancreas (not shown).

Three weeks later, he remained symptomatically anaemic, requiring further blood transfusion. Because of the history of melaena he underwent a fourth endoscopy which revealed a small, highly vascular polyp in the duodenum just beyond the angulus which bled easily on contact. Multiple biopsies were taken. The endoscopist was concerned about the possibility of a pancreatic neoplasm eroding into the duodenum and therefore an abdominal CT was arranged. Surprisingly, this demonstrated a large left renal mass with evidence of left adrenal and lung metastases, and a polypoidal mass in the medial wall of the second part of the duodenum arising from the pancreas (Figure 1). Histology from the duodenal polyp showed small, vacuolated, clear cells highlighted by immunostaining with CAM 5.2 and showing strong reactivity with Vimentin. These findings confirmed the diagnosis of metastatic RCC.

**Figure 2:**
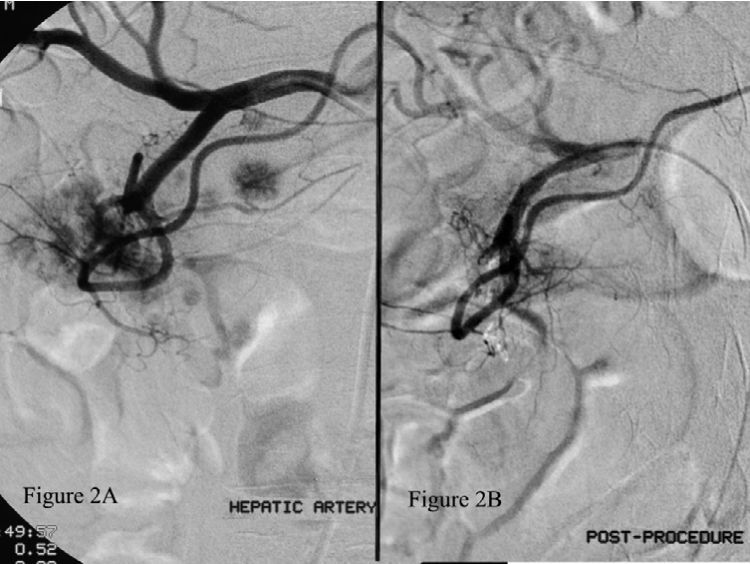
**Coeliac angiogram**. Figure 2A is an angiogram of the coeliac trunk. This demonstrates a highly vascular mass around the duodenum and pancreas and its blood supply from the pancreaticoduodenal arteries. Numerous other small vascular blushes are seen which represent further pancreatic metastases. Figure 2B is taken post- embolisation and demonstrates successful occlusion of the tumour's blood supply.

Over the three month period from initial presentation to diagnosis he required transfusion of a total of twenty units of blood for recurrent symptomatic anaemia. Given the history of recurrent melaena, coeliac angiography was undertaken with a view to trans-catheter embolisation. This was performed successfully via occlusion of the anterior and posterior pancreaticoduodenal vessels using coils (Figure 2A and 2B).

He subsequently underwent palliative left nephrectomy and histology from the resected specimen confirmed a grade 2 clear cell variant of RCC. He completed a course of interferon therapy and was able to lead a normal life for approximately 18 months, with no recurrence of anaemic symptoms or melaena. He subsequently developed painful maxillary metastases for which he received a course of radiotherapy, but died at home nearly two years after his initial presentation.

### Case 2

A 75 year old male with a past history of left nephrectomy 9 years previously for RCC was referred for out-patient investigation of iron-deficiency anaemia (Hb 10.3, MCV 78.9). Gastroscopy revealed a CLO-negative duodenal ulcer. Duodenal biopsies were within normal histological limits. Proton-pump inhibitor and iron therapy was prescribed and the patient was scheduled for review in the out-patient department.

**Figure 3:**
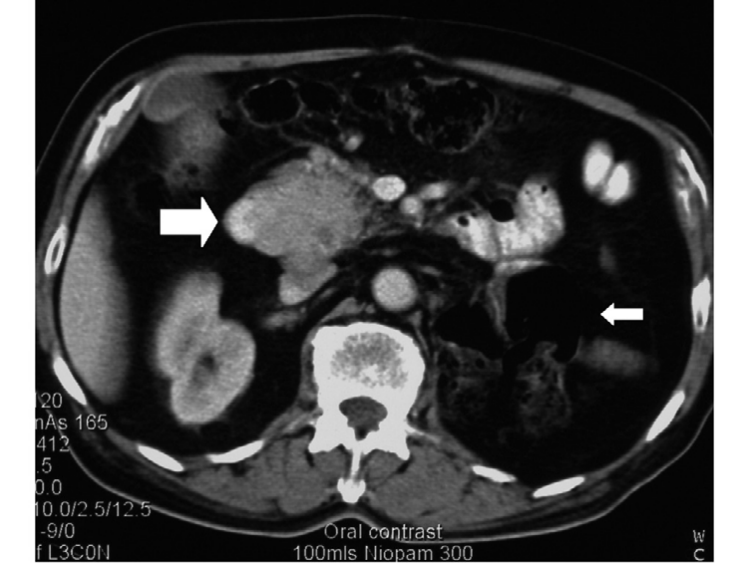
**Abdominal CT scan**. CT scan demonstrating the presence of a mass in the area of the pancreatic head and protruding into the duodenum (thick arrow) – note the previous left nephrectomy (thin arrow).

He remained anaemic three months later and repeat gastroscopy and a colonoscopy were arranged. Colonoscopy was normal, but gastroscopy revealed a fleshy vascular polyp in the duodenal bulb which was biopsied. A CT scan of the chest and abdomen, and a bone scan revealed a pancreatic mass invading the duodenum (Figure 3) but no metastatic disease elsewhere. Histology from the polyp showed multiple large clear cells which stained positively for MNF116 and Vimentin, confirming the diagnosis of metastatic RCC.

The patient was unable to tolerate interferon therapy. He became jaundiced due to tumour invasion of the distal common bile duct which was managed successfully by endoscopic biliary stenting. He received a total of 13-units of blood for symptomatic anaemia around the time metastatic disease was diagnosed, but since being commenced on a proton pump inhibitor his haemoglobin has remained stable.

## Discussion

RCC accounts for 3% of adult malignancies. It arises from the proximal tubular epithelium of the kidney and has male preponderance (M:F 2:1). Mean age at presentation is 50–70 years. Both sporadic and hereditary forms exist which are associated with genetic abnormalities on the short arm of chromosome 3 (3p). In the United Kingdom the incidence has increased by 22% over 10 years while in the United States there has been an increase of 50% in 30 years [8]. Deaths worldwide from kidney cancer exceed 100,000 per year [9].

Multiple risk factors include increased age, smoking, obesity, long-term dialysis, exposure to asbestos, petroleum products and cadmium, and several genetic syndromes including familial clear cell carcinoma, von Hippel-Lindau syndrome, and tuberous sclerosis [8].

Spread in RCC is lymphatic, haematogenous, transcoelomic, or by direct invasion. The most common sites of metastasis in RCC are the lung (75%) and lymph nodes (36%) followed by the bones (20%) and liver (18%) [9]. Pancreatic metastasis is estimated at 1.3–1.9% of autopsy series; only 50% of pancreatic metastases are symptomatic, by 1996 only 66 cases of pancreatic metastasis in RCC had been reported [10,11]. Solitary metastasis in RCC occurs in less than 5% of patients [9].

Autopsy series suggest only 2% of all tumours metastasise to the small intestine – RCC make up 7.1% of these lesions [12]. A series published by Graham [13] stated only 4% of RCC metastasize to the small intestine, while a more recent Mayo Clinic 50-year review found only 3 cases of small intestinal metastasis in RCC, which did not include cases of direct tumour extension [14].

Of all intestinal segments involved RCC metastasis to the duodenum occurs least frequently [15]. The overwhelming majority of tumours metastasising to the duodenum arise from the right kidney given its anatomic proximity [16], and of the few published reports most commonly involve the periampullary region, followed by the duodenal bulb. These usually manifest as gastrointestinal bleeding, although cases of small bowel intussusception are described [16-19]. Direct invasion of the duodenum from pancreatic metastases is also reported [13,20-22].

Gastrointestinal bleeding as the presenting symptom of a primary renal cell carcinoma is described rarely in the literature [23,24]. Bleeding is more commonly encountered in patients already known to have metastatic disease, or as the first symptom of metastatic disease in patients who have previously undergone nephrectomy for RCC [7,25,26].

Here we present a rare case of RCC whose primary manifestation of disease was with symptoms of upper gastrointestinal bleeding related to pancreatic metastases invading the duodenum. The source of bleeding in this case was obscure and initially missed by conventional gastroscopy – earlier diagnosis may have been facilitated using a side-viewing endoscope as described previously in a case of RCC metastasizing to the duodenal ampulla [27]. Bleeding in our patient was managed radiologically and without complication, by selective trans-catheter embolisation of the anterior and posterior pancreaticoduodenal arteries. Arterial embolisation to control bleeding from duodenal metastasis from RCC has been previously reported to be successful [28,29].

We also outline a case presenting 9 years after nephrectomy for RCC with isolated pancreatic and duodenal metastases causing intestinal bleeding. This is unusual as a recent study suggests the majority (83%) of tumours recur within 2 years of curative surgery, with higher recurrence rates in tumours of increasing size [30]. However, cases of late recurrence presenting as GI bleeding have been reported at 19 years after nephrectomy for RCC [31]. The longest documented survival from nephrectomy to the diagnosis of pancreatic metastasis is 27 years, and a more prolonged interval from nephrectomy to diagnosis seems to confer a better prognosis [10], presumably as a result of less aggressive tumour biology. Interestingly, in both patients described in our paper, RCC originated in the left kidney.

Palliative nephrectomy may alleviate local symptoms and can be undertaken in selected cases, but should be weighed against the burden of surgical morbidity and mortality, and is not justifiable for inducing disease regression which occurs in < 1% of patients [32]. Outcome in metastatic RCC is generally poor, with 48% 1-year and 9% 5-year survival rates. Although evidence for the role of metastasectomy in RCC is lacking [25], surgery (by duodenopancreatectomy or total pancreatectomy) should be strongly considered in especially patients with isolated pancreatic metastases where it may provide 5-year survival rates of 31–68% [33,34].

## Conclusion

Gastrointestinal bleeding from intestinal metastasis may present with overt haematemesis or signs of occult blood loss. Due to its rarity, small intestinal metastasis is often not suspected as a cause of gastrointestinal bleeding. In this report, we hope to alert clinicians to the possibility of intestinal metastasis, or local small bowel invasion from pancreatic metastasis, as a cause for gastrointestinal bleeding in patients with a past history of RCC, regardless of the time since nephrectomy. We present a rare case of RCC whose primary manifestation was with gastrointestinal bleeding. We highlight the importance of endoscopic vigilance in correctly diagnosing the source of bleeding from the upper gastrointestinal tract, and demonstrate the effective use of selective angiographic embolisation in controlling intestinal bleeding from metastatic RCC.

## Competing interests

The author(s) declare that they have no competing interests.

## Authors' contributions

GJS has drafted the manuscript. MA has reviewed the manuscript. PGW has reviewed the manuscript and was consultant in charge of care of the two patients. MSM performed the radiological investigations and angiographic embolisation and has reviewed the manuscript.

## Pre-publication history

The pre-publication history for this paper can be accessed here:
